# Revealing Metabolic Reprogramming Genes as Biomarkers of Rheumatoid Arthritis: A Bioinformatics Study Based on Ensemble Learning Approach and Single Cell RNA Sequencing

**DOI:** 10.2174/0118715303424036260226053254

**Published:** 2026-04-13

**Authors:** Lin Cheng, Hexiang Zong, Dongxu Li, Long Qian

**Affiliations:** 1 Department of Rheumatology and Immunology, The Second Affiliated Hospital of Anhui Medical University, Hefei 230031, Anhui, China

**Keywords:** Rheumatoid arthritis, metabolic reprogramming, machine learning, immune infiltration, single-cell RNA sequencing, synovial inflammation

## Abstract

**Introduction:**

Rheumatoid Arthritis (RA) is a chronic autoimmune disease characterized by persistent synovial inflammation and progressive joint destruction. Emerging evidence suggests that metabolic reprogramming plays a pivotal role in rheumatoid Arthritis (RA) pathogenesis. However, existing studies have focused predominantly on isolated pathways. This study aimed to systematically investigate the molecular interplay between metabolic reprogramming and immune dysregulation in RA.

**Methods:**

Twelve metabolism-related gene sets from MSigDB were analyzed across two bulk RNA-seq datasets (GSE93272: 232 RA/43 controls; GSE110169: 84 RA/77 controls). Based on bulk RNA-seq datasets related to RA, feature genes were selected using machine-learning algorithms, and an ensemble-learning model for RA diagnosis was developed. Subsequent analyses included Gene Set Enrichment Analysis (GSEA), immune cell infiltration profiling, drug-target prediction, and gene-disease network construction to further explore the potential functions of the feature genes. Additionally, a single-cell RNA sequencing (scRNA-seq) dataset containing a single RA patient (GSE159117) was used to identify key cell populations and investigate the interactions between cell populations. Finally, six candidate genes were validated by qPCR (RA n=5; control n=6).

**Results:**

Three high-performance diagnostic models were developed by combining the LASSO regression and XGBoost algorithms. The ensemble model (AUCs of 0.960-0.970 in training and 0.819 in external validation) integrating the 3 individual models outperformed the individual model, and 13 feature genes were identified *via* the ensemble model. Functional enrichment revealed significant associations with metabolic and immune pathways. Immune infiltration profiling revealed notably elevated T-cell subsets in RA patients, with multiple feature genes strongly correlated with T-cell activation. Single-cell analysis confirmed T cells as key mediators, with IRF7 regulating TXN and related genes. qPCR validated significant dysregulation of AKR1C3, ARG1, TXN, and C1QB in RA patients compared with controls (*p*<0.05).

**Discussion:**

These findings suggest that metabolic reprogramming is not merely a consequence but may be a driver of immune dysfunction in RA. The strong association between metabolic gene signatures and T cell activity raises the possibility that targeting metabolic pathways could modulate immune responses in RA. Further studies are needed to explore the causal relationships and therapeutic potential of these interactions in clinical settings.

**Conclusion:**

Our study provides a comprehensive characterization of metabolic-immune crosstalk in RA, identifying novel diagnostic biomarkers and therapeutic targets. Our findings advance the understanding of RA pathogenesis and may facilitate the development of precision medicine approaches.

## 1 INTRODUCTION

Rheumatoid Arthritis (RA) is a chronic autoimmune inflammatory disorder that leads to symmetrical polyarthritis, progressive joint destruction, and significant functional impairment. With a global prevalence of approximately 0.5% to 1.0%, RA represents a substantial clinical and socioeconomic burden [1]. Owing to the complicated factors contributing to its development, the precise pathogenesis of RA remains incompletely understood [2]. Although the increasing availability of various drugs has significantly improved treatments for RA, many patients experience treatment failure due to adverse effects, non-responses, or unsatisfactory treatment outcomes [3]. These challenges highlight the urgent need to elucidate RA pathogenesis and develop targeted therapeutic strategies.

Metabolic reprogramming, a hallmark of cellular adaptation, enables cells to alter their metabolic pathways to meet energy and biosynthetic demands [4]. Metabolic reprogramming involves fundamental changes in the way that cells acquire and utilize energy and biosynthetic substrates, including the regulation of multiple metabolic pathways [5]. In RA, metabolic reprogramming disrupts immune homeostasis by reshaping the behavior of immune and stromal cells within the inflamed synovium [6]. Emerging evidence suggests that cells transition from a quiescent metabolic state to a hypermetabolic phenotype, activating redox-sensitive signaling pathways and accumulating metabolic intermediates. Recent research has demonstrated that dysregulated T-cell metabolism is a central driver of autoimmune inflammation in RA. Activated T cells from RA patients display enhanced glycolysis and reduced oxidative phosphorylation, favoring effector differentiation over regulatory phenotypes [7-9]. Cai *et al*. demonstrated strong correlations between serum metabolite alterations and immune cell profiles in RA patients [10]. Notably, lactate produced by rheumatoid arthritis synovial fibroblasts modulates T-cell and macrophage differentiation and activation, further driving disease progression [11]. In addition, amino acid metabolism, particularly tryptophan degradation *via* the kynurenine pathway, modulates T-cell proliferation and tolerance [12]. Although metabolic reprogramming is increasingly recognized as a hallmark of RA, existing studies have focused predominantly on isolated pathways. A systematic investigation encompassing diverse metabolic modalities remains conspicuously absent.

Given the key role of metabolic reprogramming in RA, we hypothesize that metabolic reprogramming actively drives immune dysfunction in RA, particularly by modulating T-cell activation and differentiation. To analyze the potential genes of metabolic reprogramming, we comprehensively analyzed 12 metabolic pathways and their crosstalk in RA pathogenesis by integrating multi-omics and ensemble learning (LASSO, XGBoost, and stacking integration). This study aims to (i) identify metabolic signatures underlying RA pathogenesis, (ii) develop robust and generalizable diagnostic models, and (iii) uncover transcriptional networks linking metabolism and immune dysregulation. This multi-omics framework advances beyond prior single-pathway analyses to provide a systems-level view of metabolic-immune crosstalk in RA.

## 2 MATERIALS AND METHODS

### 2.1 Data Source

Our study obtained bulk RNA-seq data from the GEO database (https://www.ncbi.nlm.nih.gov/geo/), including the GSE93272 dataset (232 RA patients and 43 healthy controls) and the GSE110169 dataset (84 RA patients and 77 healthy controls), along with single-cell RNA sequencing (scRNA-seq) data from GSE159117 (one RA patient's blood sample). Expression data of bulk RNA-seq were normalized and batch-corrected using ComBat. To investigate metabolic reprogramming in RA, we retrieved 12 relevant gene sets from the Molecular Signatures Database (MSigDB) (Table **1**) [13] .

### 2.2 Differential Expression Analysis and Gene Set Enrichment Analysis (GSEA)

We downloaded the bulk RNA-seq datasets and extracted expression matrices *via* the R package GEOquery (v 2.72.0). Gene annotation was performed by matching platform-specific annotation files, during which we removed invalid identifiers and retained probes with the highest expression values when multiple probes mapped to the same gene. The expression matrix was log2-transformed, followed by normalization and differential expression analysis *via* the R package limma (v 1.38.3), where statistical analysis was performed using the limma package with Benjamini-Hochberg correction [14]. The significantly differentially expressed genes (DEGs) were defined as those meeting the thresholds of adjusted *p* values (adj.*P.Val*) < 0.05 and |log2FC| > 0.5. We conducted Gene Set Enrichment Analysis (GSEA) *via* the Gene Ontology (GO) and Kyoto Encyclopedia of Genes and Genomes (KEGG) pathway databases to characterize the biological functions of the DEGs.

### 2.3 Identification of Metabolic Reprogramming-Related Feature Genes

To systematically identify feature genes associated with metabolic reprogramming in RA, we integrated metabolic reprogramming-related gene sets with DEGs through a multistep analytical approach. Initially, LASSO regression combined with 10-fold cross-validation was employed to identify genes demonstrating significant contributions to the predictive model. The XGBoost algorithm was subsequently utilized to calculate the feature importance scores (gain values) for each gene, and the 5 genes with the highest contributions in each gene set (or all genes if fewer than 5 were available) were selected. Finally, on the basis of the identified feature genes, logistic regression models were constructed, and their performance was evaluated *via* area under the curve (AUC) values. The three models demonstrating superior predictive performance (highest AUC values) were selected for downstream analysis. To facilitate clinical interpretation, we developed a nomogram to visually represent the relative contribution of each gene to RA risk prediction.

### 2.4 Ensemble Model Construction *via* the Stacking-based Ensemble Learning Approach

To further improve the predictive performance, we implemented a stacking-based ensemble learning approach that combined the prediction scores (pred1, pred2, pred3) from the three top-performing models. The GSE93272 dataset was randomly divided into a training set and an internal validation set (70/30) by diagnosis. LASSO logistic regression was applied to the integrated prediction scores, and the optimal regularization parameter (Lambda) was determined through 10-fold cross-validation. The performance of the optimized ensemble model was assessed *via* Receiver Operating Characteristic (ROC) analysis of the training set, internal validation set, and external validation set (GSE110169) to evaluate its classification efficacy and stability. Comprehensive performance metrics, including accuracy, F1 score, sensitivity, and specificity, were derived from the confusion matrix analysis of the GSE110169 dataset.

### 2.5 Functional Enrichment and Subcellular Localization Analysis of Feature Genes

To investigate the biological functions and signaling pathways of the feature genes, we performed GO and KEGG enrichment analyses on the identified feature genes *via* the R package clusterProfiler (v.1.38.3) [15]. Additionally, we determined the subcellular localization of feature genes *via* the GeneCards database [16], which provided valuable insights into their potential functional roles on the basis of their distribution across cellular compartments (nucleus, cytoplasm, cell membrane, and organelles). This multifaceted analytical approach enabled systematic characterization of the biological significance of our identified feature genes in RA pathogenesis.

### 2.6 Gene‒Disease Association, Drug Prediction, and Immune Infiltration Analyses

To elucidate the broader clinical implications of the identified feature genes, we conducted comprehensive disease association and therapeutic potential analyses. Using the Comparative Toxicogenomics Database (CTD, https://ctdbase.org/), we systematically evaluated associations between feature genes and other diseases, focusing on the top 10 disease associations on the basis of inference scores and reference counts. For drug discovery, we employed DGIdb (https://dgidb.org) [17] to identify potential therapeutic compounds that target our feature genes. Molecular docking simulations were performed *via* CB-Dock2, followed by protein-drug interaction visualization *via* PyMOL to assess the binding affinities and potential mechanisms of action. To investigate the immune microenvironment in RA, single-sample gene set enrichment analysis (ssGSEA) was applied to the GSE93272 dataset to quantify the infiltration levels of 28 immune cell types. The reference gene set used for ssGSEA is listed in Table **S1**. Gene Set Variation Analysis (GSVA) was utilized to compute ssGSEA scores, and the differences between the healthy and RA groups were identified *via* Wilcoxon rank-sum tests.

### 2.7 ScRNA-seq Data Processing

The scRNA-seq data (GSE159117) of RA patients were downloaded from the GEO database and analyzed *via* the R package Seurat (v 4.3.0). Cells with >10% mitochondrial gene content, nFeature_RNA < 100 or > 4000, and nCount_RNA < 100 or > 20,000 were excluded to remove low-quality cells, empty droplets, and potential doublets. After normalization (NormalizeData function) and identification of highly variable genes (FindVariableFeatures function), dimensionality reduction was achieved through principal component analysis (top 15 PCs), followed by unsupervised clustering (FindNeighbors and FindClusters function) and UMAP visualization (RunUMAP function). The cell populations were annotated *via* established marker genes [18, 19]. To investigate intercellular crosstalk, we employed CellChat (v2.1.2) to analyze ligand-receptor interactions between the identified T-cell population and other synovial cell types, revealing key communication pathways. In addition, single-cell regulatory network inference and clustering (SCENIC) was performed to elucidate the transcriptional regulation of key cell types.

### 2.8 qPCR Validation of the Feature Genes

Total RNA was extracted from the blood samples of five RA patients and six healthy controls *via* the MolPure® Blood RNA Kit (YEASEN, Cat. 19241ES50, China). cDNA synthesis was subsequently performed *via* Hifair® III 1st strand cDNA synthesis supermix according to the manufacturer’s protocol. qPCR amplification was then conducted to specifically detect CASP3, RPS24, TXN, AKR1C3, ARG1, and C1QB expression. The PCR conditions included initial denaturation at 95°C for 30 seconds, followed by 40 cycles of 95°C for 20 seconds, 55°C for 20 seconds, and 72°C for 20 seconds. The sequences of the primers are listed in Table 2. RNA purity was assessed using a NanoDrop 2000 spectrophotometer (Thermo Fisher Scientific), and all samples exhibited A260/A280 ratios between 1.85 and 1.95. The amplification efficiencies of all target and reference genes ranged from 92% to 106%, with correlation coefficients (R^2^) ≥ 0.99. Gene expression relative to β-actin expression was determined *via* the 2^-ΔΔCt^ method.

### 2.9 Statistical Analysis

Statistical analyses were performed *via* R software (v 4.2.2) and GraphPad Prism (v 10.1.2), with network visualizations generated *via* Cytoscape (v 3.9.1). For comparisons between two groups, we employed the Student’s t test for normally distributed data and the Mann-Whitney U test for non-normally distributed data, with a *p-*value < 0.05 considered statistically significant unless otherwise specified in the results.

## 3 RESULTS

### 3.1 Construction of a Diagnostic Model Based on Metabolic Reprogramming-Related Feature Genes from an Independent Gene Set

Our study identified 457 Differentially Expressed Genes (DEGs), comprising 407 significantly upregulated genes and 50 significantly downregulated genes (Fig. **1A**). To identify the biological functions of the DEGs, GO and KEGG enrichment analyses were performed, revealing that the DEGs were enriched in metabolic or T-cell-related pathways (Figs. **1B-C**). These findings indicate that metabolic reprogramming and T cells may play critical roles in RA pathogenesis. To identify metabolic reprogramming-associated genes in RA, we performed an intersection analysis between the DEGs and genes from each gene set (Table **3**, Fig. **S1**). The glycolysis gene set was excluded from further analysis because only one overlapping gene was included.

### 3.2 **Construction and Validation of an Ensemble Diagnostic Model Based on Metabolic Reprogramming-Related Feature Genes**

Through systematic screening *via* LASSO regression and XGBoost algorithms across 11 metabolic reprogramming-related gene sets, we first identified potential feature genes *via* LASSO regression (Figs. **S2A-B**). We then evaluated their contributions to diagnostic models using XGBoost feature importance scores (Fig. **S3**). Three models for amino acid metabolism (AUC=0.960), manganese metabolism (AUC=0.957), and nucleotide metabolism (AUC=0.957) demonstrated superior diagnostic performance in the training set (Figs. **1D** and **S4A**) while maintaining robust predictive capability in the external validation cohort GSE110169 (AUC=0.686, 0.792, and 0.798, respectively; (Figs. **1E** and **S4B**). This analysis identified 13 clinically relevant feature genes (RPS24, RPL31, PSMA2, KMO, ARG1, CASP3, C1QB, TXN, AKR1C3, KYNU, HINT1, FAS, and HINT3), and three nomograms were generated (Figs. **1F** and **S4C**).

To increase model performance, we developed a stacking-based ensemble learning model integrating the three models above (amino acid metabolism, manganese metabolism, and nucleotide metabolism). The ensemble learning model demonstrated superior diagnostic performance, with AUC values of 0.970 (training set), 0.969 (internal validation set), and 0.819 (external validation set), outperforming individual models (Figs. **1G-I**, Tables **S2-3**). The nomogram based on the ensemble model revealed differential contributions of the three-component models, with the manganese metabolism model showing the strongest predictive weight (Table **4**, Fig. **1J**). The confusion matrix subsequently revealed that the model had high classification performance in the external validation set, with an accuracy of 0.78 and an F1 score of 0.75 (Fig. **1K**). Additionally, expression analysis of the feature genes revealed that all genes were upregulated in the RA group compared with the healthy group in the GSE93272 dataset and GSE110169 dataset (Figs. **S4D-E**).

### 3.3 Functional Enrichment and Subcellular Localization of 13 Feature Genes

Subcellular localization analysis of the 13 key feature genes revealed distinct compartmentalization patterns with important functional implications. All feature genes demonstrated predominant cytoplasmic and nuclear localization, suggesting potential multifunctional roles in transcriptional regulation and nucleocytoplasmic signaling (Fig. **S5**). KEGG and GO enrichment analyses of 13 feature genes revealed significant enrichment of 13 feature genes in metabolic and immune-related pathways (Figs. **2A** and **B**), indicating their potential involvement in metabolic reprogramming and immune modulation in RA. As shown in Fig. (**2A**), KEGG analysis revealed key immune- and inflammation-related pathways, including the TNF signaling pathway, p53 signaling pathway, and apoptosis pathway. GO analysis further revealed that these genes were associated with the upregulation of T-cell activation and proliferation, underscoring their roles in immune processes. Compared with those in healthy controls, immune infiltration analysis revealed elevated levels of activated CD8+ T cells, CD4+ T cells, neutrophils, macrophages, and eosinophils in RA patients (Fig. **2C**), indicating alterations in the immune microenvironment. Several feature genes exhibited strong positive correlations with T-cell subsets (Fig. **2D**). These findings indicate an association between metabolic gene expression and immune activation, although causal relationships cannot be inferred from correlation-based analyses.

### 3.4 Gene‒Disease Association and the Therapeutic Potential of the Feature Genes

Using the Comparative Toxicogenomics Database (CTD), we analyzed disease associations for the 13 feature genes. The analysis revealed significant enrichment for multiple disease categories, both of which were associated with chemical- and drug-induced liver injury, necrosis, and inflammation (Figs. **3A** and **S6**). These findings suggest shared pathogenic mechanisms between RA and these comorbid conditions. In addition, the DGIdb database identified 32 potential drugs that target feature genes (Fig. **3B**). Molecular docking of COMPOUND 24 (DGIdb identity: IUPHAR.LIGAND:10438) with the HINT1-encoded protein yielded a binding energy of -8.5 kcal/mol, with a RMSD of 1.6 Å, indicating a stable docking conformation. The ligand formed two hydrogen bonds with residues Gly105 and ASN99 (Fig. **3C**). The drug prediction analysis results revealed ligand binding *via* hydrogen bonds to protein residues, suggesting potential impacts on protein function and gene regulation.

### 3.5 Quality Control, Clustering, and Cell Type Annotation of scRNA-seq Data

In total, 10,495 cells were obtained from the GSE159117 dataset, and 10,161 cells were retained after quality control (Fig. **4A**). Following the identification of the top 4,000 highly variable genes and principal component analysis, we detected 18 distinct clusters (Figs. **4B-C** and **S7A-B**). The top 5 marker genes in each cluster are presented in the heatmap (Fig. **4D**). Using well-established marker genes (Fig. **S7C**, Table **S4**), the clusters were subsequently annotated into seven cell types (Fig. **4E**): monocytes, dendritic cells (DCs), plasmacytoid dendritic cells (pDCs), T cells, natural killer (NK) cells, B cells, and plasmablasts. Notably, T cells represented the predominant population (Fig. **S7D**). Finally, we investigated the expression levels of the selected genes shown in (Figs. **4F** and **S7E**).

### 3.6 Analysis of Cell‒Cell Communication in the RA Microenvironment

To explore interactions among different cell populations, we performed CellChat analysis to construct a cell-cell communication network. Notably, T cells emerged as the central hub in the RA microenvironment, exhibiting the greatest number and strength of interactions (Fig. **5A**). Specifically, T cells showed stronger interactions with NK cells, monocytes, and pDCs (Fig. **5B**). Signaling pattern analysis revealed that MHC-I signaling dominated both the incoming and outgoing pathways in T cells (Figs. **5C** and **D**). Given these findings, we further investigated the MHC-I pathway across different cell types. The results demonstrated that MHC-I signaling was primarily sent by various cell types but was predominantly received by T cells (Fig. **5E**). The strongest ligand‒receptor interactions involved HLA-A-CD8B, HLA-B-CD8B, and HLA-C-CD8B, with CD8A and CD8B (the key receptors) being specifically expressed in T cells (Figs. **5F** and **5G**). Our study elucidates the critical role of T-cell interactions in the RA microenvironment and highlights the MHC-I pathway as a potential therapeutic target through the modulation of its ligand‒receptor pairs.

### 3.7 Transcriptional Regulatory Network Analysis Reveals Key Transcription Factors Associated with T Cells in the RA Immune Microenvironment

Given the potential role of T cells in RA, we investigated Transcription Factors (TFs) and their target genes within T-cell populations *via* SCENIC. The heatmap illustrates the regulon activity of the TFs regulating selected feature genes (Fig. **6A**). Additionally, we constructed a regulatory network to visualize interactions between feature genes and their associated TFs (Fig. **6B**, Table **S5**). Our analysis identified IRF7 as a central transcription factor with the highest connectivity regulating multiple target genes, including KMO, CASP3, RPL31, FAS, RPS24, AKR1C3, TXN, C1QB, KYNU, and HINT1. Among these genes, TXN exhibited the highest degree of coregulation and was targeted by several TFs, which suggests its involvement in RA pathogenesis. Bulk transcriptomic analysis further revealed that IRF7 expression was significantly elevated in RA patients compared with healthy controls in the bulk RNA-seq dataset (Fig. **6C**, *p* < 0.01). Moreover, in the scRNA-seq dataset, IRF7 showed high regulon activity scores, particularly within T-cell populations, indicating transcriptional activation at the single-cell level.

### 3.8 qPCR Validation

We further selected the six genes with the greatest contributions to the ensemble learning model and validated their expression levels by comparing the mRNA abundance between RA patients and healthy controls. Consistent with the RNA-seq results above, the expression levels of ARG1, TXN, C1QB, RPS24, and PSMA2 were elevated in the RA group (Figs. **7A-E**). However, the differences in RPS24 and PSMA2 were not statistically significant. In contrast, AKR1C3 expression was downregulated in the RA samples (Fig. **7F**). These results support the reliability of our bioinformatic predictions and highlight these feature genes as potential therapeutic targets for RA.

## 4 DISCUSSION

Metabolic reprogramming, a critical adaptive mechanism enabling cells to respond to pathological conditions, plays a pivotal role in immune regulation and inflammatory responses in RA [7-9]. However, existing studies have focused predominantly on isolated pathways. In this work, we comprehensively analyzed 12 metabolic pathways and their crosstalk in RA pathogenesis. Using a stacking-based ensemble learning approach, we developed an optimized diagnostic model to predict RA progression. Moreover, we elucidated the biological functions of these genes in RA-mediated metabolic reprogramming.

Given the proven efficacy of machine learning in clinical diagnostics [20], we employed XGBoost and LASSO regression to construct three RA diagnostic models on the basis of distinct metabolic reprogramming gene sets. Furthermore, an ensemble model was developed on the basis of a stacking ensemble learning approach, which was composed of 13 feature genes (RPS24, RPL31, PSMA2, KMO, ARG1, CASP3, C1QB, TXN, AKR1C3, KYNU, HINT1, FAS, and HINT3). Previous studies have demonstrated that functional enzymes, including AKR1C3, CASP3, KYNU, PSMA2, and ARG1, are closely associated with RA progression by modulating the immune microenvironment [21-25]. Additionally, the ribosomal proteins RPS24 and RPL31 cause immunosuppression in RA through protein synthesis in peripheral blood immune cells [26]. C1QB, a pivotal component of the classical complement pathway, plays a significant role in diverse immune regulatory processes [27]. Emerging evidence highlights the diagnostic potential and clinical relevance of C1QB in RA, with strong correlations observed across multiple immune cell populations [28]. FAS and TXN induce apoptosis and mediate fibroblast-like synoviocyte death, thereby inhibiting the development of RA [29, 30]. Notably, this study is the first to identify KMO, HINT1, and HINT3 as RA-associated factors. The protein products of KMO have been implicated in inflammatory pathways [31], whereas HINT1 is involved in fundamental biological processes, including transcriptional regulation, cell cycle progression, and apoptosis [32]. Furthermore, HINT3 has been detected in dendritic cells and linked to mitochondrial dysfunction, as evidenced by its role as a biomarker in periodontitis patients [33]. Collectively, these findings suggest that KMO, HINT1, and HINT3 may contribute to RA pathogenesis *via* immunomodulation and metabolic disruption.

The ensemble learning model was developed on the basis of three metabolic pathways: manganese metabolism, amino acid metabolism, and nucleotide metabolism. Manganese and other nutrient elements play crucial roles in regulating inflammation and the immune system [34-36]. A previous study demonstrated that manganese enhances nitric oxide production by increasing L-arginine levels, thereby exacerbating inflammatory reactions in Rheumatoid Arthritis (RA) patients [37]. Additionally, disruptions in amino acid metabolism can influence RA progression. Notably, tryptophan (Trp) metabolism is substantially altered in RA, which strongly correlates with disease severity [38]. The tryptophan-kynurenine pathway, mediated primarily by indoleamine 2,3-dioxygenase (IDO1), regulates T-cell differentiation and immune tolerance by depleting tryptophan and generating immunosuppressive kynurenine metabolites [12]. Dysregulation of this pathway in RA has been linked to a breakdown of immune tolerance and enhanced effector T-cell activation. In parallel, nucleotide metabolism contributes to inflammation through altered purine and pyrimidine turnover. Increased ATP release and adenosine receptor signaling can modulate macrophage polarization and cytokine secretion, while excessive nucleotide catabolism supports the high-energy demand of activated immune cells [39].

Enrichment analyses of the identified feature genes revealed their predominant associations with biological processes, including negative regulation of activated T-cell proliferation and the Trp metabolism pathway. Immune infiltration analysis further demonstrated significant correlations between these genes and key immune cell populations, particularly activated CD8+ T cells and activated CD4+ T cells. In support of this, a mouse model of autoimmune gastritis showed that silencing the KMO gene exacerbated disease severity, likely due to impaired kynurenine metabolism, which promotes Th17 cell differentiation and proinflammatory IL-17 production [40]. While our immune infiltration profiling highlights a close association between metabolic reprogramming and T-cell activity, these results are correlative. Further mechanistic and experimental studies, such as *in vitro* modulation of key metabolic genes or *in vivo* immune perturbation models, are required to establish causality.

ScRNA sequencing data revealed that T cells serve as a pivotal cell population in RA, which has been demonstrated in previous studies [41, 42]. SCENIC analysis further revealed that IRF7 may act as the core TF governing T-cell functionality in RA, with the highest regulatory degree among target genes, particularly thioredoxin (TXN). IRF7, a well-characterized TF in the IRF family, drives type I interferon programs that heighten antigen presentation and costimulation, while indirectly increasing cellular oxidative load. Recent studies implicate its role in connective tissue diseases, including Systemic Lupus Erythematosus (SLE) and rheumatoid arthritis [43, 44]. In arthritis models, IRF7 deficiency exacerbates clinical severity and increases the levels of proinflammatory cytokines, suggesting its potential as an RA biomarker [44]. The TXN gene reduces oxidative constraints on TCR signaling, enabling sustained activation in a ROS-rich environment. Truncated thioredoxin (Txn80) promotes monocyte/macrophage activation and IL-12 secretion, suggesting that it is involved in Th1-driven immune responses and IFN-γ production [45]. Notably, TXN is upregulated in RA and has been proposed as a potential diagnostic biomarker [46]. Our findings highlight a potential IRF7-TXN regulatory axis that bridges immune activation and redox homeostasis in T cells. This interaction aligns with recent studies demonstrating that metabolic stress and mitochondrial dysfunction in RA T cells lead to aberrant ROS accumulation, which in turn reinforces inflammatory signaling [9]. A schematic model summarizing the proposed IRF7-TXN-metabolic regulatory axis and its role in sustaining T-cell activation and synovial inflammation in RA is shown in Fig. (**8**).

Our integrative multi-omics and machine-learning analysis highlights several metabolic signatures with potential diagnostic utility for RA. The identified feature genes, particularly KMO, HINT1, HINT3, and TXN, may serve as promising biomarkers to support early detection or risk stratification. Moreover, the metabolic pathways implicated in this study, including the kynurenine pathway and redox-regulatory networks, present potential therapeutic targets for modulating aberrant immune activation in RA.

While these findings offer valuable insights into RA pathogenesis, several limitations should be acknowledged. Firstly, although the predictive model was validated *via* an independent dataset, its generalizability requires further confirmation through large-scale, multicenter clinical studies. Secondly, the qPCR verification was performed on a relatively small sample size (n = 11), which warrants cautious interpretation and requires expansion in future work. Thirdly, the current scRNA-seq analysis was derived from a single sample, and expanding validation to additional clinical specimens is essential to strengthen the reliability of these observations. Additionally, the molecular mechanisms underlying the identified biomarkers warrant further *in vitro* and *in vivo* experimental validation in future investigations.

## CONCLUSIONS

The present study revealed that 13 metabolic reprogramming-related genes were associated with RA, and for the first time, three feature genes, KMO, HINT1, and HINT3, were discovered. Immune infiltration analysis highlighted the central role of T cells in RA pathogenesis, and SCENIC-based regulatory network analysis revealed that IRF7 is a core transcription factor with TXN as its most connected target gene. Through computational drug prediction and molecular docking, potential therapeutic targets and candidate drugs were identified, offering new insights for precision medicine in RA.

## Figures and Tables

**Fig. (1):**
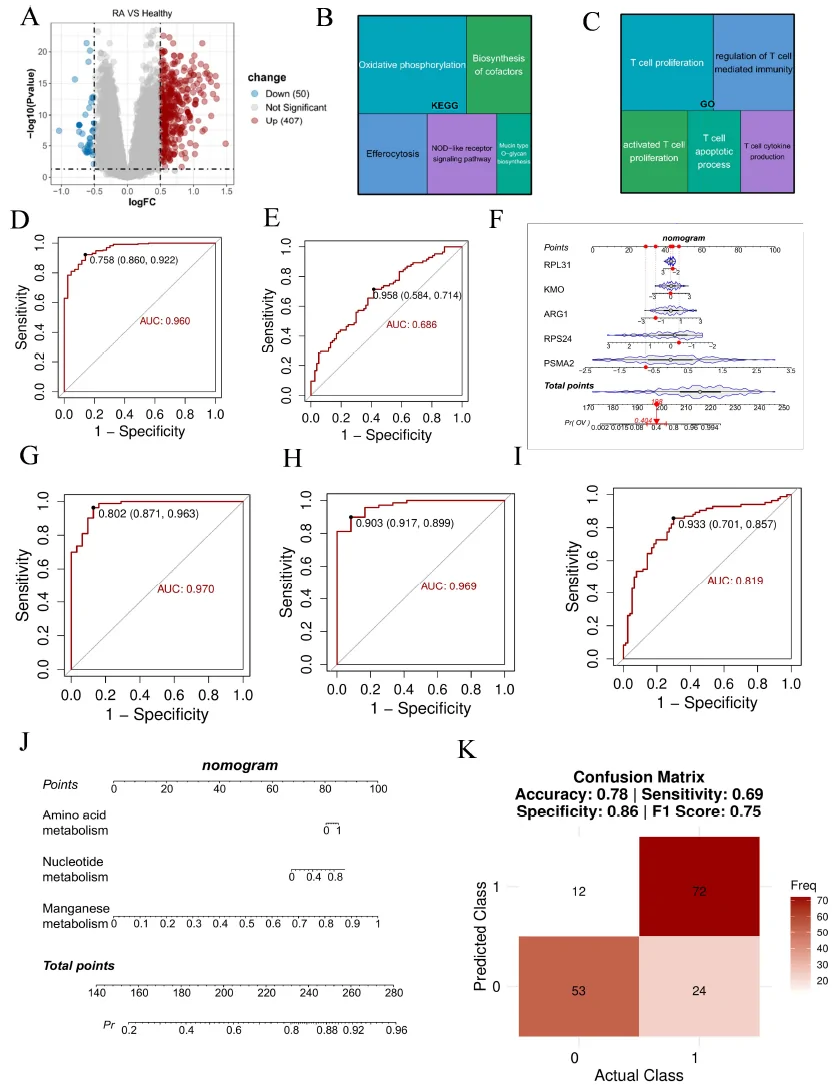
Construction and validation of the stacking-based ensemble learning model based on amino acid, manganese, and nucleotide metabolism. (**A**) Volcano plot of DEGs in RA (n = 232 RA and n = 43 controls). (**B**) KEGG enrichment analysis of DEGs. (**C**) GO enrichment analysis of DEGs. (**D-E**) ROC curves for the training (**D**) and external validation (**E**) sets of the amino acid metabolism-based model. (**F**) Nomogram for the ensemble learning model of amino acid metabolism. (**G-I**) ROC curves for the training (**G**), internal validation (**H**), and external validation (**I**) sets of the stacking-based ensemble learning model. (**J**) Nomogram of the ensemble learning model. (**K**) Confusion matrix showing the classification performance of the ensemble model in the validation set.

**Fig. (2):**
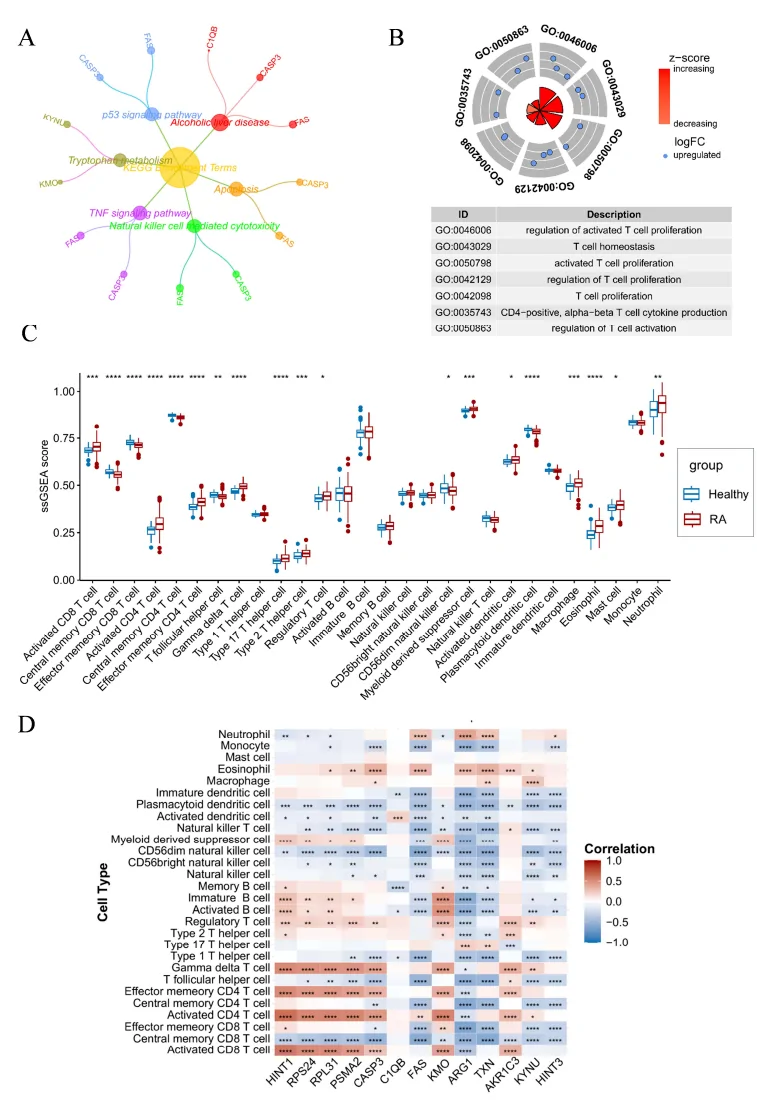
Functional enrichment analysis and immune infiltration analysis of feature genes. (**A**) KEGG enrichment analysis. (**B**) GO enrichment analysis. (**C**) Boxplots showing the enrichment scores of 28 immune cell types in the RA and healthy groups. (**D**) Heatmap of Spearman correlations between immune cell enrichment scores and feature gene expression. Correlations were assessed using Spearman’s rank test. *p < 0.05, **p < 0.01, ***p < 0.001, ****p < 0.0001.

**Fig. (3):**
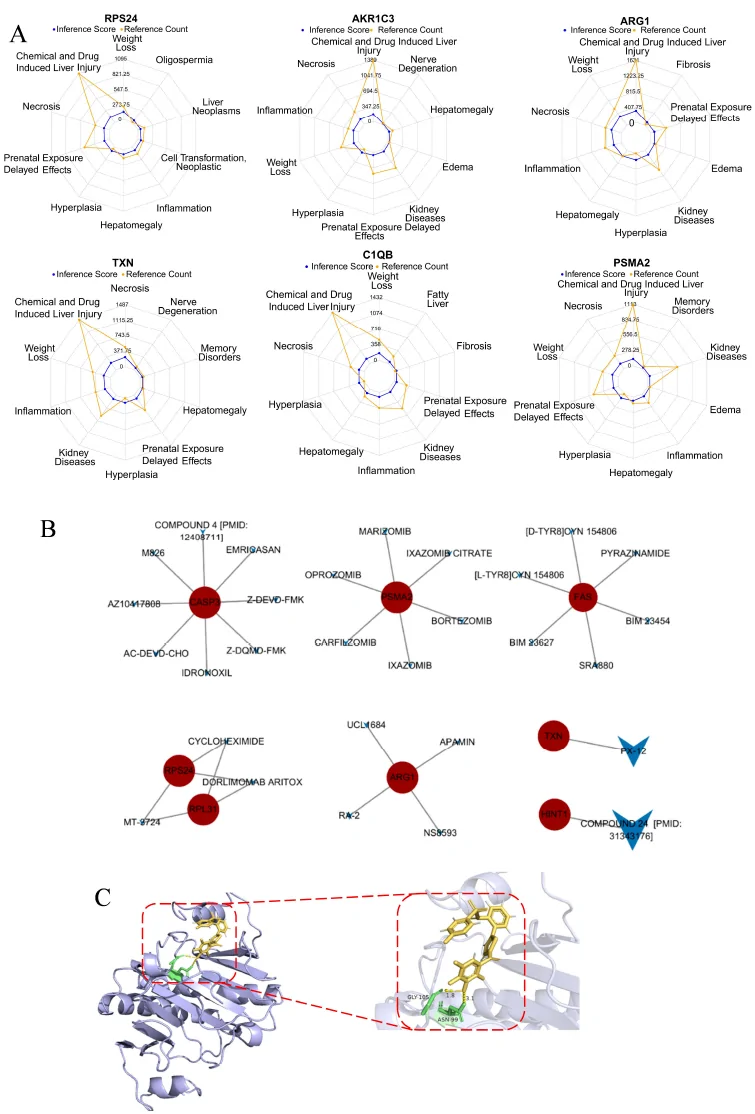
Disease and drug prediction. (**A**) Disease prediction of RPS24, AKR1C3, ARG1, TXN, C1QB, and PSMA2. (**B**) Network of predicted drug‒gene interactions. Red nodes represent RA feature genes, and blue nodes represent candidate drugs. (**C**) Molecular docking results for COMPOUND 24 and the HINT1-encoded protein. The global (left) and local (right) views of the docking structure are shown, with hydrogen bonds highlighted in yellow.

**Fig. (4):**
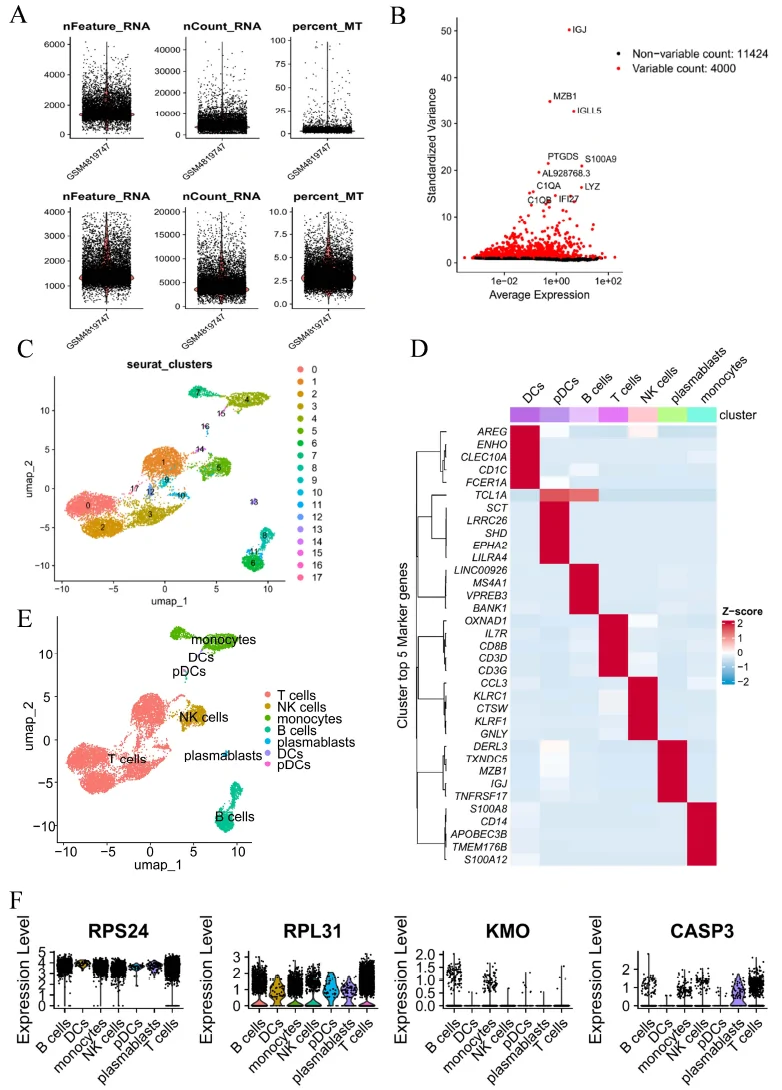
Analysis of the scRNA-seq data. (**A**) nFeature_RNA, nCount_RNA, and percent_MT before (upper) and after (bottom) quality control. (**B**) Variance plot showing 15,424 genes in all cells, and the red dots represent the top 4000 highly variable genes. (**C**) UMAP plot showing the 18 cell clusters. (**D**) Heatmap depicting the expression of the top 5 marker genes among the seven cell populations. (**E**) UMAP plot showing the annotation of each cell type. (**F**) Violin diagrams showing the expression levels of RPS24, RPL31, KMO, and CASP3 in different cell populations.

**Fig. (5):**
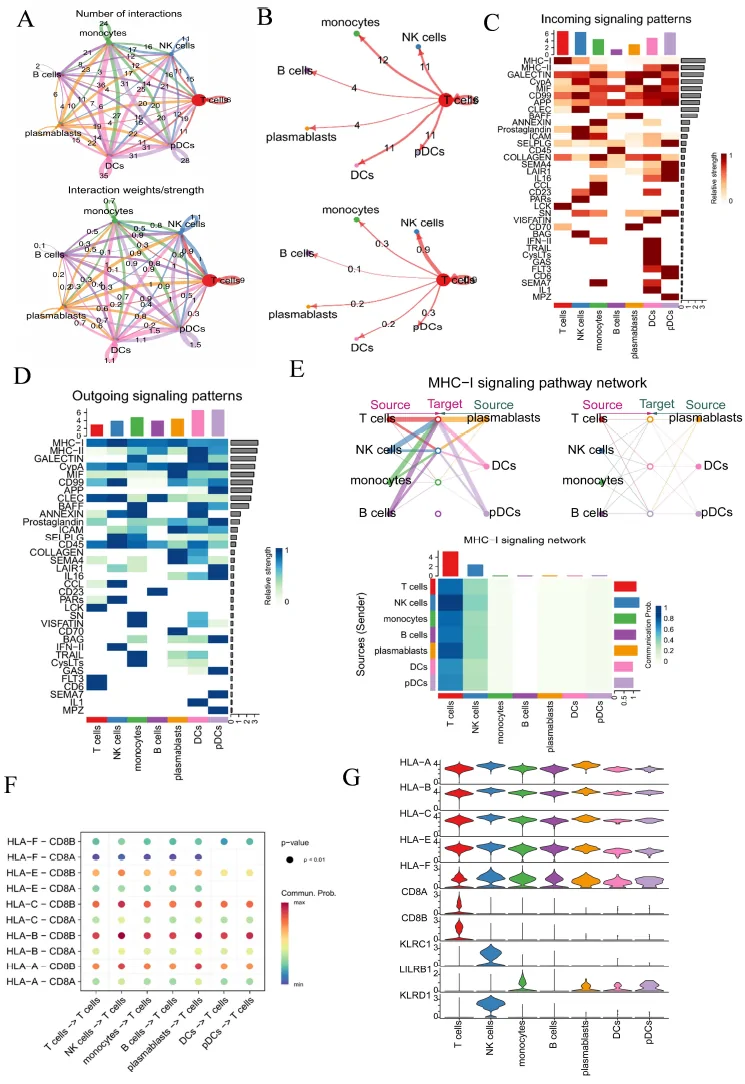
Cell-cell communication in RA. (**A**) Circle plots showing the number (upper) and strength (lower) of interactions between cells. (**B**) Circle plots showing the number (upper) and strength (lower) of interactions between T cells and other cell types as sources. (**C-D**) Heatmap displaying the patterns identified in the incoming (**C**) and outgoing (**D**) signaling pathways. (**E**) Visualization of the MHC-I signaling pathway. (**F**) Bubble plot showing the probability of ligand‒receptor interaction. (**G**) Violin diagrams showing the expression of ligand receptors in seven cell populations.

**Fig. (6):**
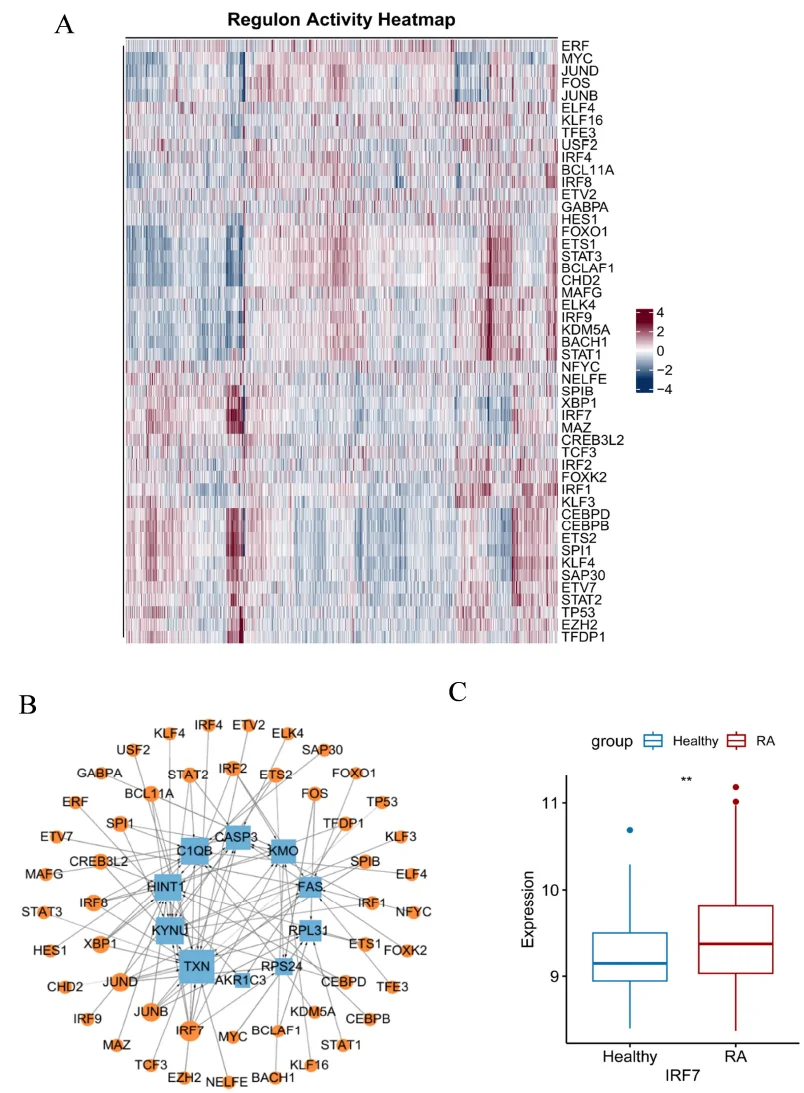
Transcription factor regulatory network in T cells. (**A**) Heatmap showing the regulatory effects of TFs on feature genes. (**B**) Network of key genes and corresponding TFs. The node size represents the degree of connectivity. (**C**) Boxplot showing the expression of IRF7 in bulk RNA-seq data between RA patients and healthy controls. **p < 0.01.

**Fig. (7):**
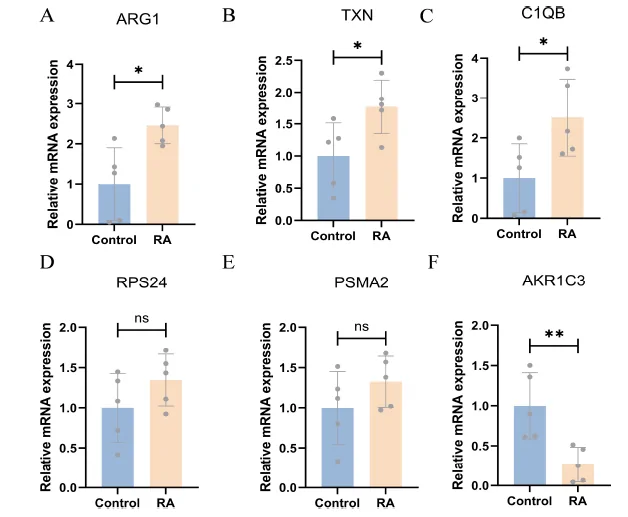
qPCR validation. The mRNA expression levels of ARG1 (**A**), TXN (**B**), C1QB (**C**), RPS24 (**D**), PSMA2 (**E**), and AKR1C3 (**F**) (n=5 RA and n=6 control). Statistical analysis of qPCR results was performed using the Student’s t-test. *p < 0.05, **p < 0.01.

**Fig. (8):**
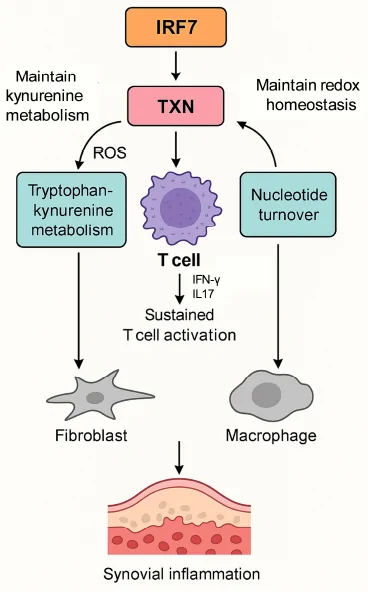
Proposed IRF7-TXN-metabolic regulatory network in RA.

**Table 1:** Metabolic reprogramming-related gene sets.

Gene Set	Gene Number
Amino Acid Metabolism	374
Cholesterol Metabolism	140
Fatty Acid Biosynthetic Process	166
Fatty Acid Metabolism	158
Lactate Metabolism	333
Lipid Metabolism	776
Manganese Metabolism	1399
Nucleotide Metabolism	882
Oxidative Phosphorylation	200
Purine Metabolism	159
Pyrimidine Metabolism	98
Glycolysis	74

**Table 2:** List of qPCR primers.

Gene	Sequence
human-CASP3-F	GAAATTGTGGAATTGATGCGTGA
human-CASP3-R	CTACAACGATCCCCTCTGAAAAA
human-RPS24-F	ATGAACGACACCGTAACTATCCG
human-RPS24-R	CCGAATTTCTGTCTTAGGCACTG
human-TXN-F	GTGAAGCAGATCGAGAGCAAG
human-TXN-R	CGTGGCTGAGAAGTCAACTACTA
human-AKR1C3-F	TCTGGGATCTCAACGAGACAA
human-AKR1C3-R	TGGAACTCAAAAACCTGCACG
human-ARG1-F	TGGACAGACTAGGAATTGGCA
human-ARG1-R	CCAGTCCGTCAACATCAAAACT
human-C1QB-F	TTCTGTGACTATGCCTACAACAC
human-C1QB-R	5'-GCCCAGTAGTGAGTTCTTGTC
human-β-actin-F	5'-CATGTACGTTGCTATCCAGGC
human-β-actin-R	5'-CTCCTTAATGTCACGCACGAT

**Table 3:** Number of intersecting genes between each gene set and DEGs.

Gene Sets	Number of Intersection Genes
Amino acid metabolism	29
Cholesterol metabolism	6
Fatty acid biosynthetic process	5
Fatty acid metabolism	3
Lactate metabolism	10
Lipid metabolism	15
Manganese metabolism	34
Nucleotide metabolism	20
Oxidative phosphorylation	17
Purine metabolism	3
Pyrimidine metabolism	4
Glycolysis	1

**Table 4:** Coefficients of genes in each model.

Variants	Amino Acid Metabolism	Manganese Metabolism	Nucleotide Metabolism	Stacking Model
Intercept	3.9135872	4.8723685	4.1120197	-1.6616334
RPS24	-2.8809363	-	-	-
RPL31	-0.3616254	-	-	-
PSMA2	4.7510645	-	-	-
KMO	0.8527391	-	-	-
ARG1	1.3681525	-		-
CASP3	-	0.7157020	-	-
C1QB	-	2.2054436	-	-
TXN	-	1.4282515	1.0121517	-
AKR1C3	-	0.8472188	1.2594350	-
KYNU	-	1.0673194	-	-
HINT1	-	-	0.4718037	-
FAS	-	-	1.2107554	-
HINT3	-	-	1.1556504	-
pred1	-	-	-	0.1676830
pred2	-	-	-	3.6274130
pred3	-	-	-	0.7344128

**Note:** “-” indicates that the variant is not used in the model.

## Data Availability

All RNA-sequencing and single-cell sequencing data from rheumatoid arthritis patients were obtained from the Gene Expression Omnibus (GEO) database (https://www.ncbi.nlm.nih.gov/geo/query/acc.cgi).

## LIST OF ABBREVIATIONS

RA: Rheumatoid arthritis
scRNA-seq: Single-cell RNA sequencing
GSEA: Gene Set Enrichment Analysis
AUC: Area Under the Curve
CTD: Comparative Toxicogenomics Database
GSVA: Gene Set Variation Analysis
SCENIC: Single-Cell Regulatory Network Inference and Clustering
DEGs: Differentially Expressed Genes
LASSO: Least Absolute Shrinkage and Selection Operator
XGBoost: eXtreme Gradient Boosting

## STANDARDS OF REPORTING

STROBE guidelines were followed.
