# Supplementary material for: Revealing Metabolic Reprogramming Genes as Biomarkers of Rheumatoid Arthritis: A Bioinformatics Study Based on Ensemble Learning Approach and Single Cell RNA Sequencing
Source: Endocr Metab Immune Disord Drug Targets. 2026 Apr 13;26:E18715303424036. doi: 10.2174/0118715303424036260226053254 (PMC13670193; doi:10.2174/0118715303424036260226053254)
Supplement: Supplementary file 1 [file EMIDDT-26-E18715303424036_SD1.pdf]

Supplementary Material

Revealing Metabolic Reprogramming Genes as Biomarkers of Rheumatoid Arthritis: A Bioinformatics Study Based on Ensemble Learning Approach and Single Cell RNA Sequencing

Lin Cheng[1], Hexiang Zong[1], Dongxu Li[1] and Long Qian[1,*]

[1]*Department of Rheumatology and Immunology, The Second Affiliated Hospital of Anhui Medical University, Hefei 230032, Anhui, China*

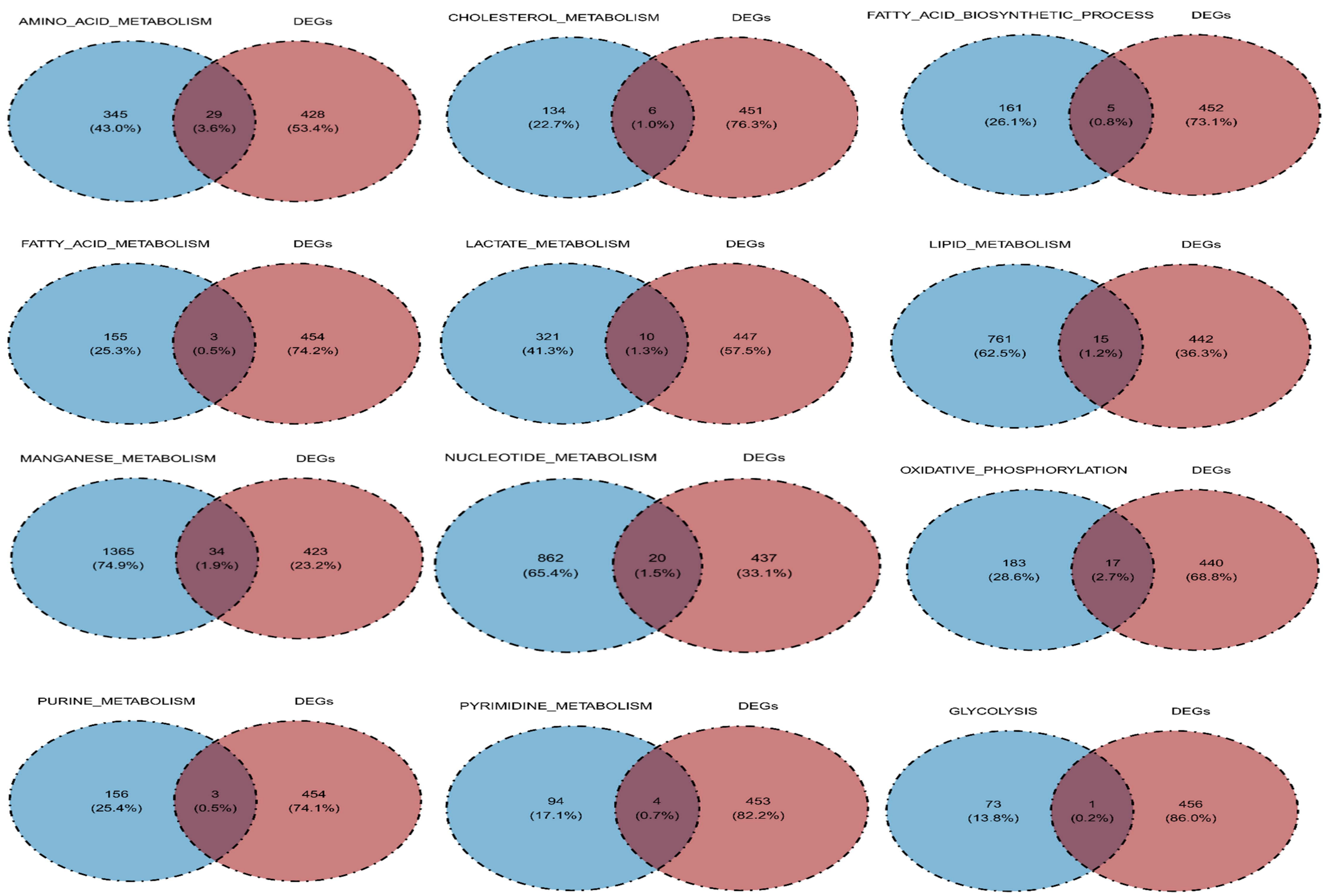

Fig. S1.

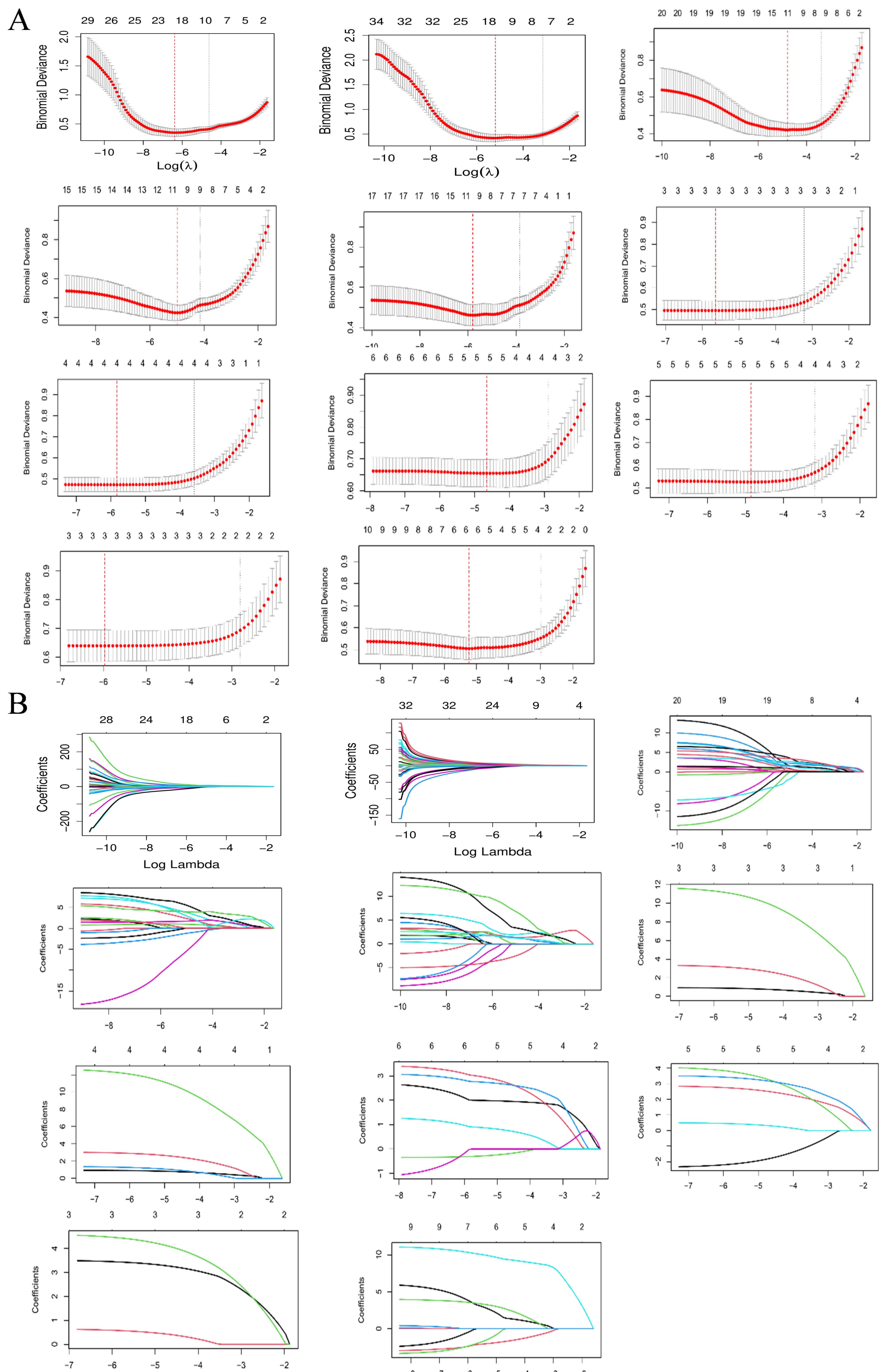

Fig. S2.

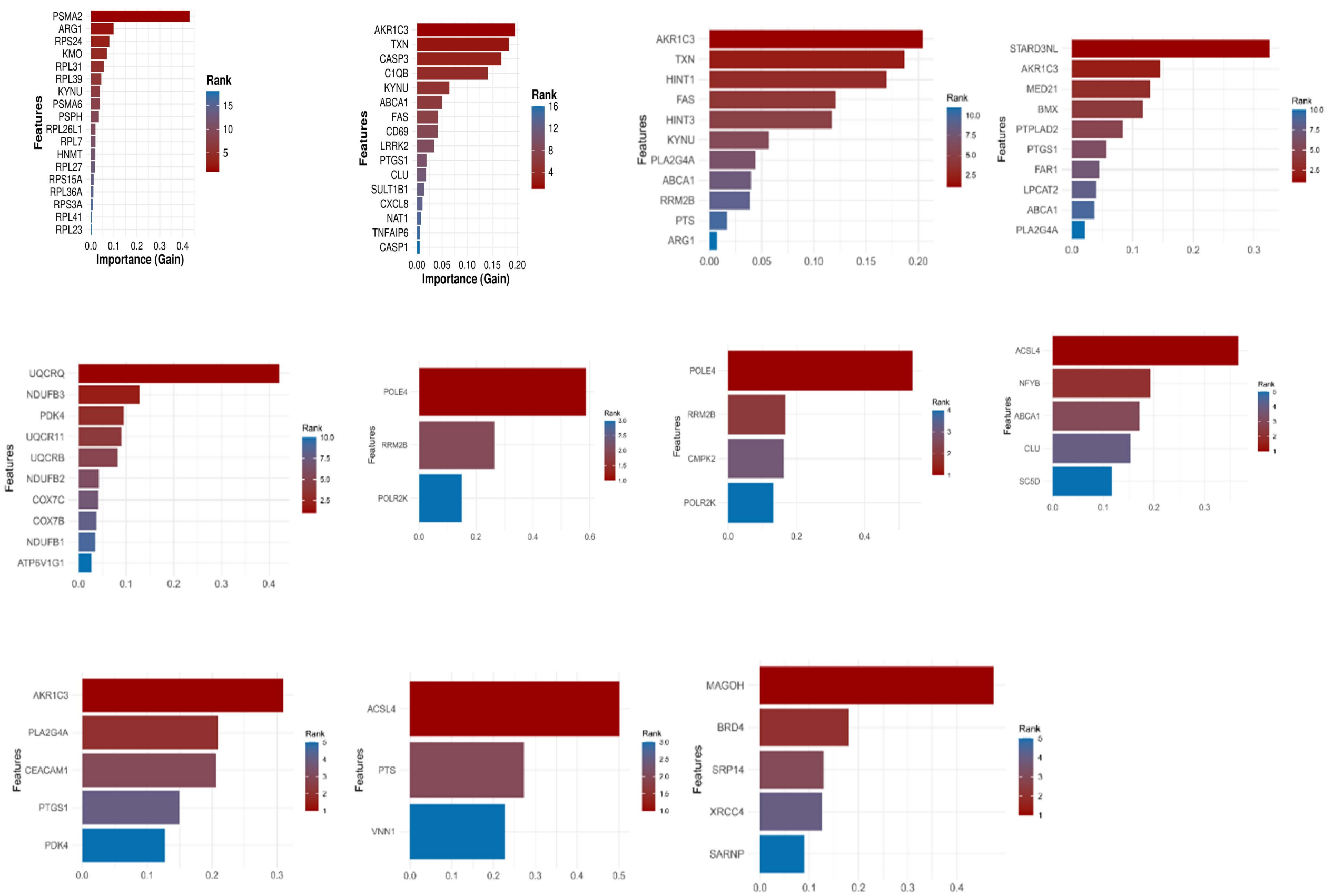
PSMA2
ARG1
RPS24
KMO
RPL31
RPL39
KYNU
PSMA6
PSPH
RPL26L1
RPL7
HNMT
RPL27
RPS15A
RPL36A
RPS3A
RPL41
RPL23
Features
Importance (Gain)
Rank
AKR1C3
TXN
CASP3
C1QB
KYNU
ABCA1
FAS
CD69
LRRK2
PTGS1
CLU
SULT1B1
CXCL8
NAT1
TNFAIP6
CASP1
HINT1
HINT3
PLA2G4A
RRM2B
PTS
STARD3NL
MED21
BMX
PTPLAD2
FAR1
LPCAT2
UQCRQ
NDUFB3
PDK4
UQCR11
UQCRB
NDUFB2
COX7C
COX7B
NDUFB1
ATP6V1G1
POLE4
POLR2K
CMPK2
ACSL4
NFYB
SC5D
PLA2G4A
CEACAM1
VNN1
MAGOH
BRD4
SRP14
XRCC4
SARNP

Fig. S3

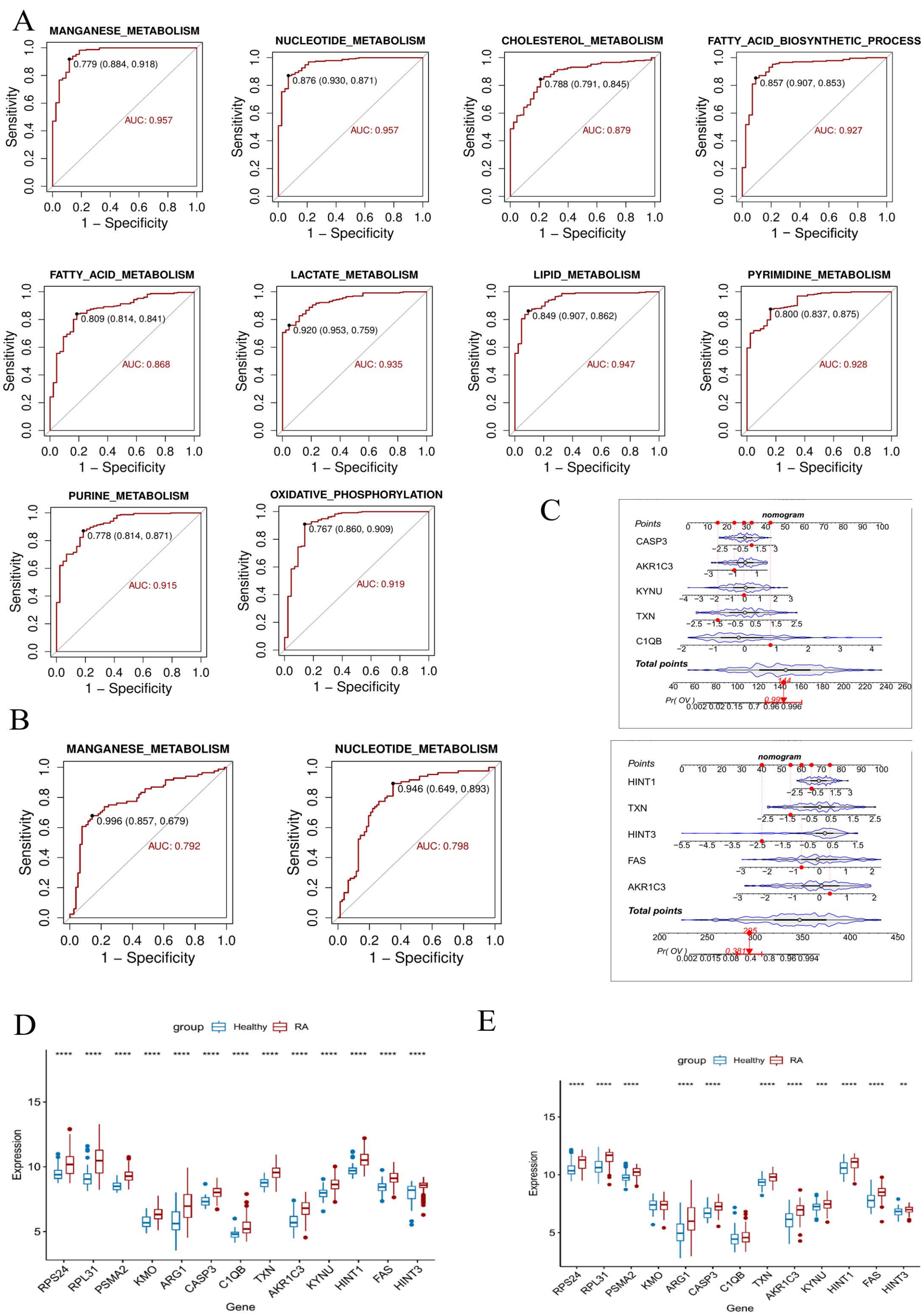
A
MANGANESE_METABOLISM
0.779 (0.884, 0.918)
AUC: 0.957
NUCLEOTIDE_METABOLISM
0.876 (0.930, 0.871)
AUC: 0.957
CHOLESTEROL_METABOLISM
0.788 (0.791, 0.845)
AUC: 0.879
FATTY_ACID_BIOSYNTHETIC_PROCESS
0.857 (0.907, 0.853)
AUC: 0.927
FATTY_ACID_METABOLISM
0.809 (0.814, 0.841)
AUC: 0.868
LACTATE_METABOLISM
0.920 (0.953, 0.759)
AUC: 0.935
LIPID_METABOLISM
0.849 (0.907, 0.862)
AUC: 0.947
PYRIMIDINE_METABOLISM
0.800 (0.837, 0.875)
AUC: 0.928
PURINE_METABOLISM
0.778 (0.814, 0.871)
AUC: 0.915
OXIDATIVE_PHOSPHORYLATION
0.767 (0.860, 0.909)
AUC: 0.919
Sensitivity
1 – Specificity
B
MANGANESE_METABOLISM
0.996 (0.857, 0.679)
AUC: 0.792
NUCLEOTIDE_METABOLISM
0.946 (0.649, 0.893)
AUC: 0.798
C
nomogram
Points
CASP3
AKR1C3
KYNU
TXN
C1QB
Total points
Pr(OV)
nomogram
Points
HINT1
TXN
HINT3
FAS
AKR1C3
Total points
Pr(OV)
D
group Healthy RA
Expression
Gene
RPS24 RPL31 PSMA2 KMO ARG1 CASP3 C1QB TXN AKR1C3 KYNU HINT1 FAS HINT3
E
group Healthy RA
Expression
Gene
RPS24 RPL31 PSMA2 KMO ARG1 CASP3 C1QB TXN AKR1C3 KYNU HINT1 FAS HINT3

Fig. S4.

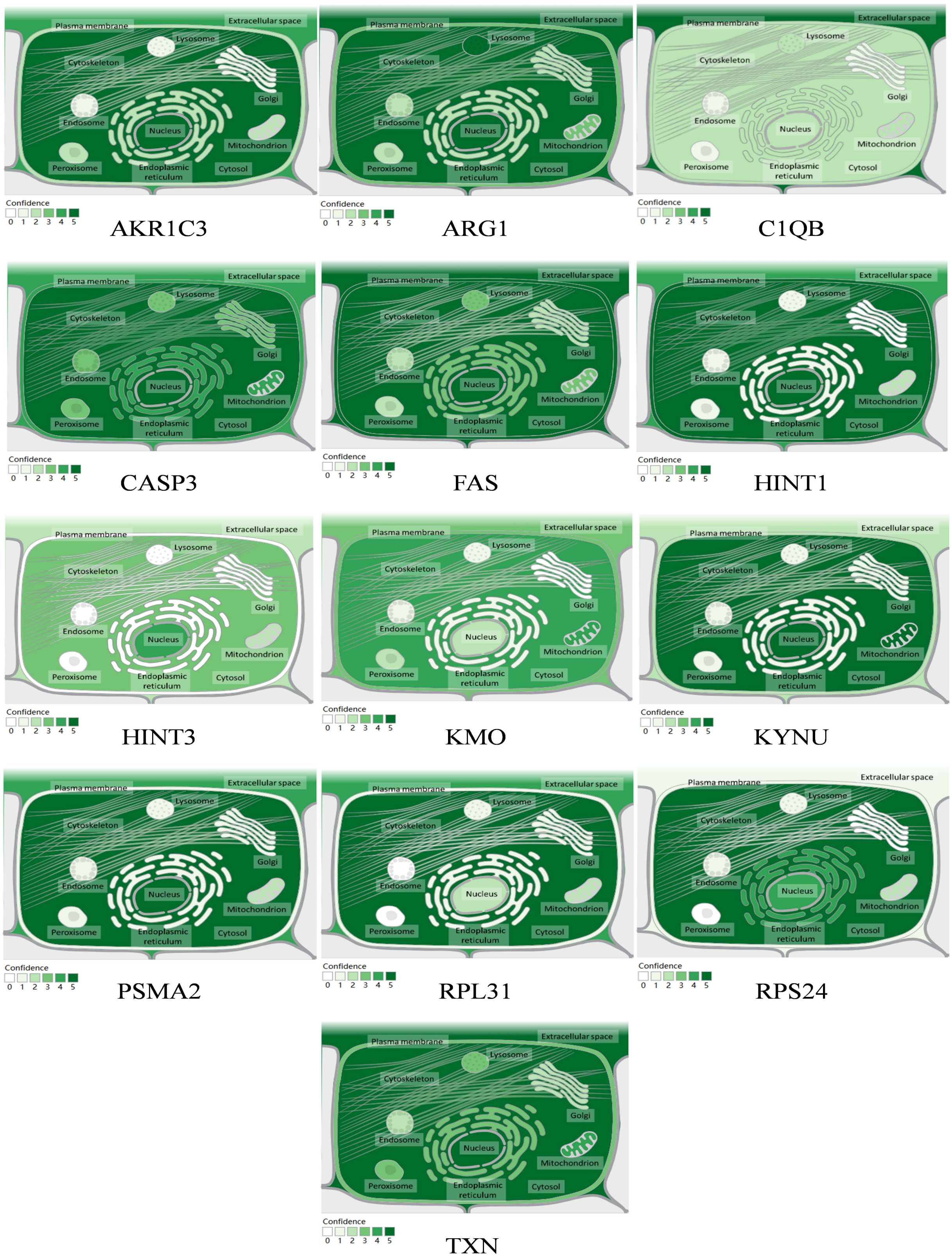
Plasma membrane
Extracellular space
Lysosome
Cytoskeleton
Golgi
Endosome
Nucleus
Mitochondrion
Peroxisome
Endoplasmic reticulum
Cytosol
Confidence
0 1 2 3 4 5
AKR1C3
ARG1
C1QB
CASP3
FAS
HINT1
HINT3
KMO
KYNU
PSMA2
RPL31
RPS24
TXN

Fig. S5.

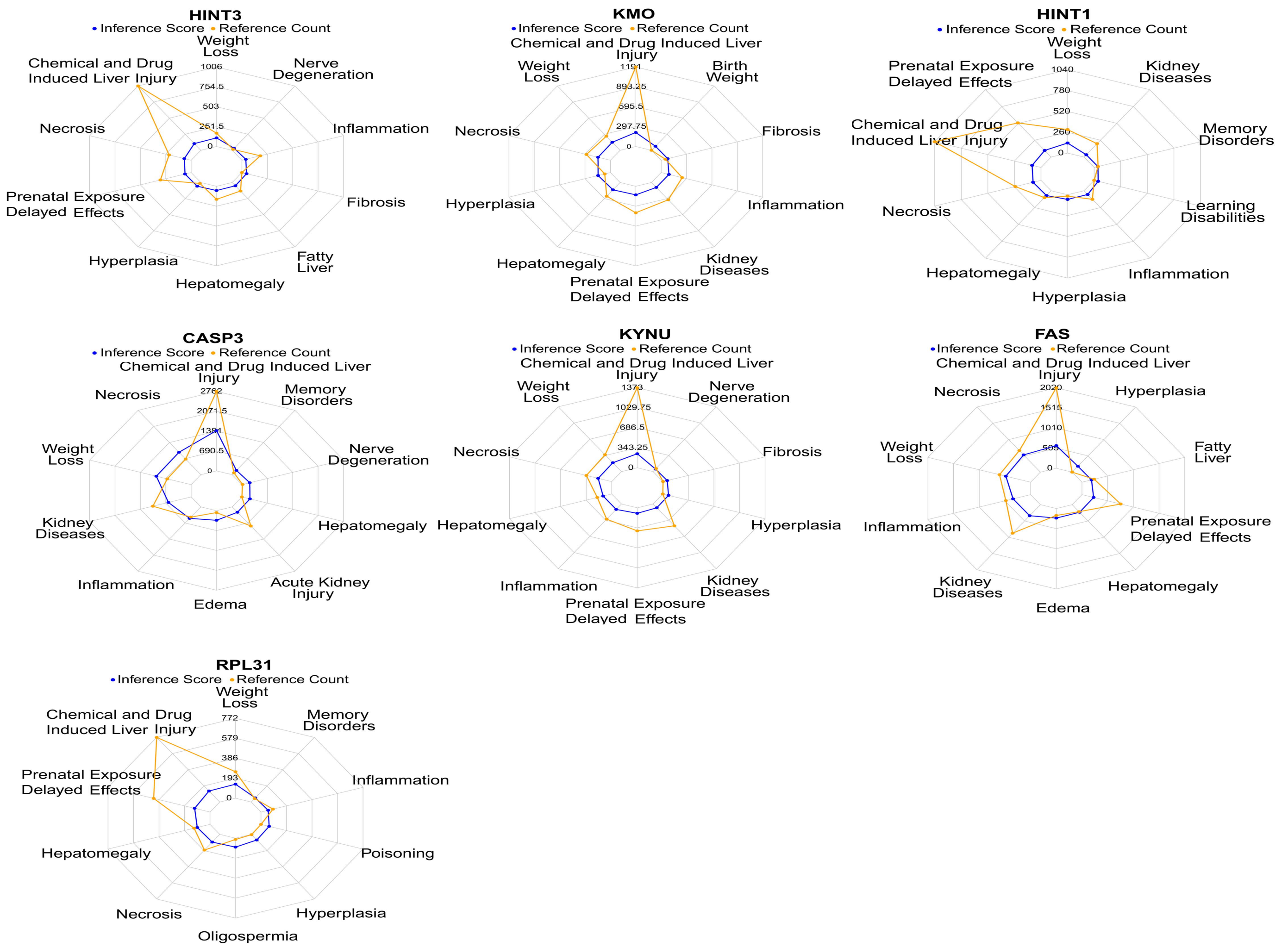
HINT3
Inference Score
Reference Count
Weight Loss
Nerve Degeneration
Inflammation
Fibrosis
Fatty Liver
Hepatomegaly
Hyperplasia
Prenatal Exposure Delayed Effects
Necrosis
Chemical and Drug Induced Liver Injury
1006
754.5
503
251.5
0
KMO
Inference Score
Reference Count
Chemical and Drug Induced Liver Injury
Birth Weight
Fibrosis
Inflammation
Kidney Diseases
Prenatal Exposure Delayed Effects
Hepatomegaly
Hyperplasia
Necrosis
Weight Loss
1191
893.25
595.5
297.75
0
HINT1
Inference Score
Reference Count
Weight Loss
Kidney Diseases
Memory Disorders
Learning Disabilities
Inflammation
Hyperplasia
Hepatomegaly
Necrosis
Chemical and Drug Induced Liver Injury
Prenatal Exposure Delayed Effects
1040
780
520
260
0
CASP3
Inference Score
Reference Count
Chemical and Drug Induced Liver Injury
Memory Disorders
Nerve Degeneration
Hepatomegaly
Acute Kidney Injury
Edema
Inflammation
Kidney Diseases
Weight Loss
Necrosis
2762
2071.5
1381
690.5
0
KYNU
Inference Score
Reference Count
Chemical and Drug Induced Liver Injury
Nerve Degeneration
Fibrosis
Hyperplasia
Kidney Diseases
Prenatal Exposure Delayed Effects
Inflammation
Hepatomegaly
Necrosis
Weight Loss
1373
1029.75
686.5
343.25
0
FAS
Inference Score
Reference Count
Chemical and Drug Induced Liver Injury
Hyperplasia
Fatty Liver
Prenatal Exposure Delayed Effects
Hepatomegaly
Edema
Kidney Diseases
Inflammation
Weight Loss
Necrosis
2020
1515
1010
505
0
RPL31
Inference Score
Reference Count
Weight Loss
Memory Disorders
Inflammation
Poisoning
Hyperplasia
Oligospermia
Necrosis
Hepatomegaly
Prenatal Exposure Delayed Effects
Chemical and Drug Induced Liver Injury
772
579
386
193
0

Fig. S6.

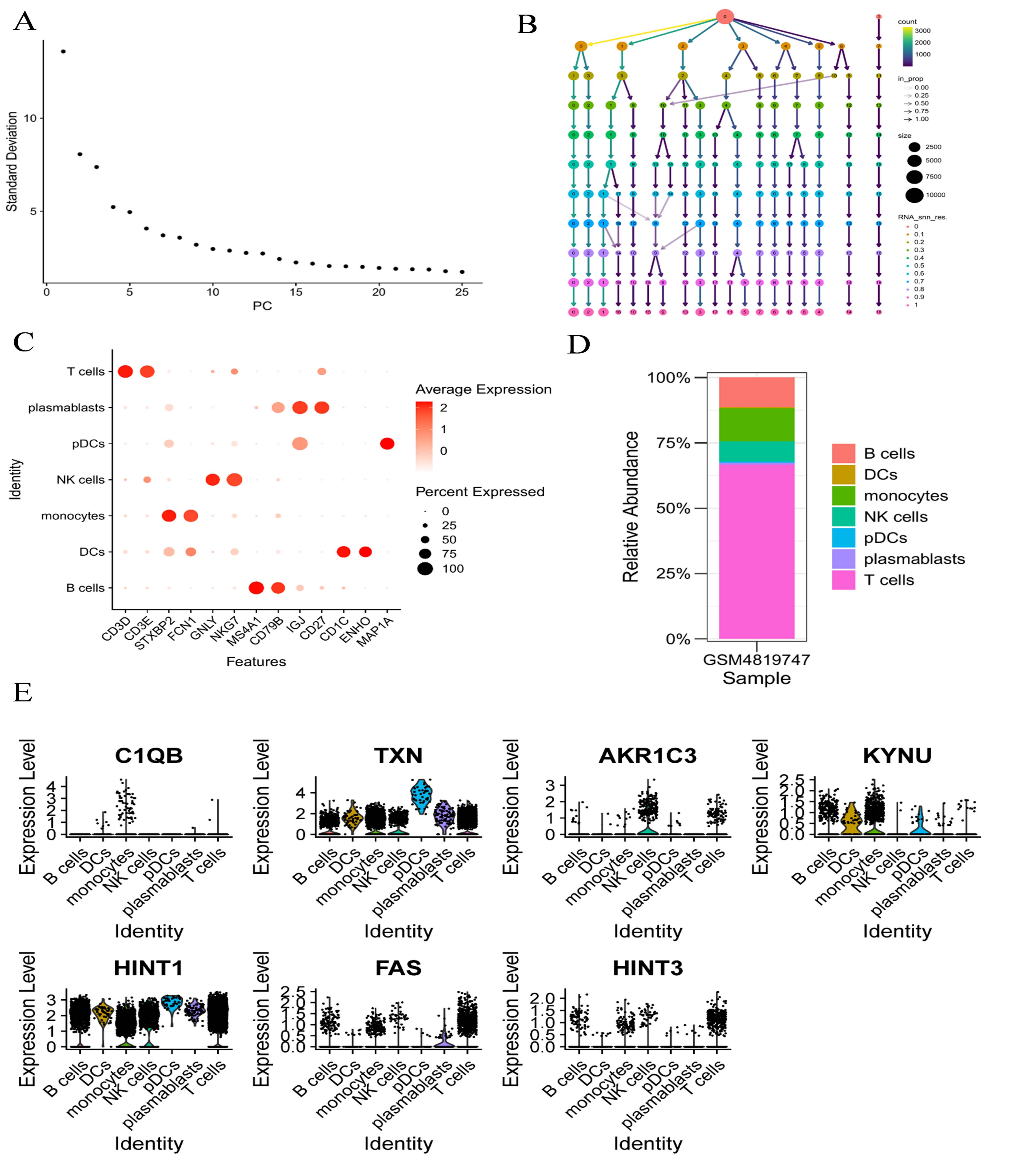
A
Standard Deviation
PC
B
count
in_prop
size
RNA_snn_res.
C
Identity
T cells
plasmablasts
pDCs
NK cells
monocytes
DCs
B cells
CD3D CD3E STXBP2 FCN1 GNLY NKG7 MS4A1 CD79B IGJ CD27 CD1C ENHO MAP1A
Features
Average Expression
Percent Expressed
D
Relative Abundance
GSM4819747
Sample
B cells
DCs
monocytes
NK cells
pDCs
plasmablasts
T cells
E
C1QB
TXN
AKR1C3
KYNU
HINT1
FAS
HINT3
Expression Level
Identity

Fig. S7.

STROBE Statement—checklist of items that should be included in reports of observational studies

	Item No.	Recommendation	Page No.	Relevant text from manuscript
**Title and abstract**	1	(*a*) Indicate the study's design with a commonly used term in the title or the abstract	1	Revealing Metabolic Reprogramming Genes as Biomarkers of Rheumatoid Arthritis: A Bioinformatics Study Based on Ensemble Learning Approach and Single Cell RNA Sequencing
		(*b*) Provide in the abstract an informative and balanced summary of what was done and what was found		Methods: Twelve metabolism-related gene sets from MSigDB …. Finally, six candidate genes were validated by qPCR (RA n=5; control n=6).<br><br>Results: Three high-performance diagnostic models were developed by combining the LASSO regression and XGBoost algorithms. The ensemble model … TXN and related genes. qPCR validated significant dysregulation of AKR1C3, ARG1, TXN, and C1QB in RA patients compared with controls ($p<0.05$).
**Introduction**				
Background/rationale	2	Explain the scientific background and rationale for the investigation being reported	2	Rheumatoid arthritis (RA) is a chronic autoimmune disease characterized by synovial inflammation and progressive joint destruction… Metabolic reprogramming has been increasingly recognized as an important driver in immune cell activation and inflammatory responses
Objectives	3	State specific objectives, including any prespecified hypotheses	3	In this study, we aimed to identify metabolism-related diagnostic biomarkers of RA and develop a robust machine-learning diagnostic model by integrating bulk transcriptomic data and single-cell RNA sequencing analysis.
**Methods**				
Study design	4	Present key elements of study design early in the paper	3	Two bulk transcriptome datasets were used for identifying differentially expressed metabolic genes and constructing diagnostic models, and scRNA-seq was used to validate the expression patterns and immune-related mechanisms
Setting	5	Describe the setting, locations, and relevant dates, including periods of recruitment, exposure, follow-up, and data collection	3	Bulk expression profiles were obtained from GEO datasets: GSE93272 and GSE110169. Single-cell dataset GSE159117 was used for scRNA-seq analysis.
Participants	6	a) ***ohort study***— Give the eligibil-	4	GSE93272 included 84 RA patients and 77 healthy controls… scRNA-seq dataset GSE159117 included synovial tissue cells from one RA patient and one healthy control… quality control thresholds: genes detected in

		ity criteria, and the sources and methods of selection of participants. Describe methods of follow-up<br>b) ***ase-control study***—Give the eligibility criteria, and the sources and methods of case ascertainment and control selection. Give the rationale for the choice of cases and controls<br>c) ***ross-sectional study***—Give the eligibility criteria, and the sources and methods of selection of participants		200–3000 cells and mitochondrial gene percentage < 10%
		d) ***ohort study***—For matched studies, give matching criteria and number of exposed and unexposed<br>*e)* ***ase-control study***—For matched studies, give matching criteria and the number of controls per case		Not applicable

Variables	7	Clearly define all outcomes, exposures, predictors, potential confounders, and effect modifiers. Give diagnostic criteria, if applicable		The diagnostic outcome was RA vs. healthy control. Predictor variables included metabolism-related gene expression levels… key genes selected for the final diagnostic model were C1QB, IRF7, and PTGER4.
Data sources/ measurement	8*	For each variable of interest, give sources of data and details of methods of assessment (measurement). Describe comparability of assessment methods if there is more than one group	4-6	Differential expression analysis was performed using limma… metabolism-related gene sets were obtained from MSigDB… scRNA-seq data were processed using Seurat, and immune infiltration was estimated by ssGSEA
Bias	9	Describe any efforts to address potential sources of bias	3	Expression data of bulk RNA-seq were normalized and batch-corrected using ComBat
Study size	10	Explain how the study size was arrived at		Not applicable

Quantitative variables	11	Explain how quantitative variables were handled in the analyses. If applicable, describe which groupings were chosen and why	5-7	Differential expression: limma; machine learning: LASSO with 10-fold cross-validation, XGBoost feature ranking; model performance: ROC curves, AUC, confusion matrix metrics; immune infiltration: ssGSEA and Wilcoxon test; scRNA-seq analysis: Seurat; cell communication: CellChat; transcription factor analysis: SCENIC.
Statistical methods	12	(*a*) Describe all statistical methods, including those used to control for confounding	4,7	Genes with adjusted P value < 0.05 and |log2FC| > 0.5 were considered differentially expressed… expression matrices were normalized before model training.<br><br>The Statistical analysis section in Page 7
		(*b*) Describe any methods used to examine subgroups and interactions		Not explicitly described; GEO datasets used are preprocessed expression matrices.
		(*c*) Explain how missing data were addressed		Not explicitly described; GEO datasets used are preprocessed expression matrices
		(*d*) *Cohort study*—If applicable, explain how loss to follow-up was addressed<br><br>*Case-control study*—If applicable, explain how		Not applicable (public GEO transcriptomic datasets; no matching design described)

		matching of cases and controls was addressed<br><br>*Cross-sectional study*—If applicable, describe analytical methods taking account of sampling strategy		
		(*e*) Describe any sensitivity analyses		
**Results**				
Participants	13*	(a) Report numbers of individuals at each stage of study—eg numbers potentially eligible, examined for eligibility, confirmed eligible, included in the study, completing follow-up, and analysed	3	GSE93272 contained 84 RA and 77 healthy controls; scRNA-seq dataset GSE159117 contained synovial cells from one RA patient and one healthy control.
		(b) Give reasons for non-participation at each stage		Not applicable
		(c) Consider use of a flow diagram		Not included as a standard flow diagram
Descriptive data	14*	(a) Give characteristics of study participants (eg demographic, clinical, social) and information on exposures and potential confounders		Not all baseline clinical characteristics are provided; samples were described by GEO dataset group labels (RA vs control)
		(b) Indicate number of participants with missing data for each variable of interest		Not reported
		(c) *Cohort study*—Summarise follow-up time (eg, average and total amount)		
Outcome data	15*	*Cohort study*—Report numbers of outcome events or summary measures over time	8	The diagnostic model discriminated RA and controls with AUC values reported in ROC analysis (training/testing/external validation).
		Case-control study—Report numbers in each exposure category, or		

		summary measures of exposure		
		Cross-sectional study—Report numbers of outcome events or summary measures		
Main results	16	(*a*) Give unadjusted estimates and, if applicable, confounder-adjusted estimates and their precision (eg, 95% confidence interval). Make clear which confounders were adjusted for and why they were included	8	ROC-AUC results were reported (e.g., ensemble model AUC ~0.90 in training/testing and ~0.82 in external validation). Confidence intervals were not explicitly reported.
		(*b*) Report category boundaries when continuous variables were categorized		Not applicable
		(*c*) If relevant, consider translating estimates of relative risk into absolute risk for a meaningful time period		
Other analyses	17	Report other analyses done—eg analyses of subgroups and interactions, and sensitivity analyses	7,9,10,11	Functional enrichment (GO/KEGG), immune infiltration comparison, scRNA-seq cell-type expression validation, cell-cell communication analysis (CellChat), transcription factor regulatory network analysis (SCENIC).
**Discussion**				
Key results	18	Summarise key results with reference to study objectives	12,13	We identified three metabolism-related diagnostic biomarkers (C1QB, IRF7, PTGER4) and constructed an ensemble machine-learning diagnostic model with good predictive performance
Limitations	19	Discuss limitations of the study, taking into account sources of potential bias or imprecision. Discuss both direction and magnitude of any potential bias	14	Limitations include small sample size for experimental validation and scRNA-seq dataset containing only one RA and one control sample, which may affect generalizability
Interpretation	20	Give a cautious overall interpretation of results considering objectives, limitations, multiplicity of	14	Our findings suggest that metabolic pathways and immune-related mechanisms contribute to RA pathogenesis, and the identified genes may be potential diagnostic biomarkers and therapeutic targets.

		analyses, results from similar studies, and other relevant evidence		
Generalisability	21	Discuss the generalisability (external validity) of the study results	14	External validation in an independent dataset demonstrated stable diagnostic performance; however, further validation in larger cohorts is needed.
**Other information**				
Funding	22	Give the source of funding and the role of the funders for the present study and, if applicable, for the original study on which the present article is based	15	This study was supported by funding sources as listed in the Funding section.

***Give information separately for cases and controls in case-control studies and, if applicable, for exposed and unexposed groups in cohort and cross-sectional studies.**
